# *In Vitro* Interactions of Extracellular Histones with LDL Suggest a Potential Pro-Atherogenic Role

**DOI:** 10.1371/journal.pone.0009884

**Published:** 2010-03-25

**Authors:** Alan D. Pemberton, Jeremy K. Brown

**Affiliations:** The Roslin Institute and Royal (Dick) School of Veterinary Studies, University of Edinburgh, Roslin, Midlothian, United Kingdom; Massachusetts Institute of Technology, United States of America

## Abstract

**Background:**

Nuclear histones have previously been shown to aggregate LDL *in vitro*, suggestive of a possible pro-atherogenic role. Recent studies indicate that histones are released during acute inflammation, and therefore might interact with circulating lipoproteins *in vivo*. In view of the associative link between inflammation and cardiovascular disease, the behaviour of histones was investigated using *in vitro* models of LDL retention and foam cell formation.

**Methodology/Principal Findings:**

Heparin agarose beads were used as a model of a matrix rich in sulphated glycosaminoglycans, to which histones bind strongly. Histone-modified beads were observed to pull down more LDL from solution than untreated beads, indicating that histones can function as bridging molecules, enhancing LDL retention. Furthermore, addition of heparin inhibited histone-induced aggregation of LDL. To model foam cell formation, murine RAW 264.7 macrophages were incubated for 24 h in the presence of LDL, histones, LDL plus histones or vehicle control. Cells incubated with LDL in the presence of histones accumulated significantly more intracellular lipid than with LDL or histone alone.

**Conclusions/Significance:**

These results are consistent with a potential pro-atherogenic role for extracellular histones, which should be investigated further.

## Introduction

Nuclear histones are highly abundant, small, basic proteins that serve to package DNA in the nucleosome, as demonstrated by X-ray crystallography[1]. The predominant histones are the linker histone H1, and the core histones H2A, H2B, H3 and H4. In terms of physical characteristics, histones are relatively small proteins (11–21 kDa), rich in basic amino acid residues.

Histones and other polycationic proteins are known to bind strongly to polyanions, most notably the highly sulphated glycosaminoglycan, heparin. Their affinity for heparin is stronger than that for DNA, such that heparin is able to solubilise histones from isolated nuclei[2]. Histones also demonstrate a strong affinity for binding anionic phospholipids such as phosphatidylserine and cardiolipin[3].

Histones induce plasma proteins to form aggregates, of which fibrinogen has been identified as a major component[4]. By applying proteomics to further characterise this aggregate, we recently discovered[5] that apolipoproteins form a significant part of such aggregates, indicating the participation of lipoproteins. The interactions of histones with lipoproteins have not previously been studied in detail, with the exception of the work of Skrzydlewski[6], who in the 1970s reported the formation of aggregates of LDL in the presence of histones, and hypothesised that this phenomenon might contribute to the development of atherosclerosis[7]. Intriguingly, the detection of histone H2A in HDL fractions has been claimed in a patent (US 2007/0099242 A1) to be useful as a biomarker of cardiovascular disease.

In this study therefore, we sought to confirm the aggregation of LDL by histones, and further investigate the relevance of histones to atherosclerosis, through the use of *in vitro* models of LDL retention and foam cell generation.

## Methods

### Aggregation of low-density lipoprotein with histones

Human low-density lipoprotein (LDL; density 1.019–1.063 g/ml; 5 mg/ml total protein, Intracel, Frederick, MD, USA) was diluted with PBS to 1.67 mg/ml total protein, and any pre-existing aggregates were removed by centrifugation (10 min at 20,000 g, 15°C). Aliquots (75 µl) of diluted LDL were mixed with 25 µl of various dilutions of calf thymus histones (Sigma, Poole, Dorset, UK) in PBS, and incubated at room temperature for 40 min. Turbidity was then measured at 680 nm. The aggregation of LDL in the presence of histones was also observed over time (180 sec), by absorbance at 680 nm, following addition of 25 µl of 0, 0.5, 1 and 2 mg/ml histones in PBS to 75 µl of 1.33 mg/ml LDL in PBS.

### Aggregation of high-density lipoprotein with histones

High density lipoprotein (HDL; density 1.063–1.21 g/ml; 20 mg/ml total protein; Intracel, Frederick, MD, USA) was diluted to 2 mg/ml by protein in PBS and centrifuged at 20,000 g for 10 min at 15°C. Samples of diluted HDL (50 µl) were mixed with histones (50 µl of 0, 0.2, 0.4, 0.6, 0.8 and 1 mg/ml histones in PBS), incubated at room temperature for 40 min, then turbidity measured at 680 nm. In another experiment, histones (50 µl of 0, 0.5 and 1 mg/ml in PBS) were added to 50 µl of HDL (2 mg/ml in PBS) and absorbance at 680 nm was measured over the first 180 sec.

### Inhibition of histone-induced LDL aggregation using heparin

Histones (25 µl of 1 mg/ml in PBS) were added to a mixture of LDL (65 µl of 1.92 mg/ml in PBS) and 10 µl of unfractionated heparin (UFH, Sigma) or low molecular weight heparin (LMWH, Sigma) (both at 0, 0.01, 0.1, 1 or 10 mg/ml in PBS). After incubation for 1 hr at room temperature, turbidity was measured by absorbance at 680 nm.

### Binding of LDL and HDL to histone-loaded heparin agarose beads

Heparin agarose suspension (100 µl, Sigma) was pre-incubated in spin columns (VectaSpin Micro, 10 µm, Whatman International Ltd., Maidstone, UK) with 100 µl of 0, 1, 2 or 4 mg/ml calf thymus histones in PBS. After washing with PBS, the matrix was incubated with 100 µl of LDL solution (0.5 mg/ml total protein in PBS) or 100 µl of HDL (1 mg/ml total protein in PBS). The unbound lipoprotein was recovered following a pulse spin, and the spin column was then washed twice with 500 µl of PBS before elution with 100 µl of non-reducing SDS-PAGE sample buffer. Equal volumes of unbound and eluted material were then analysed by SDS-PAGE, revealing ApoB100 bands (LDL) or ApoA1 bands (HDL), which were quantified by gel densitometry (Image J; http://rsbweb.nih.gov/ij/). In the case of the HDL experiment, additional lipoprotein-free controls were run to allow densitometry correction for histone H1 protein in the eluted samples, which runs at the same MW as ApoA1 by SDS-PAGE.

### Histone-induced LDL uptake by RAW macrophages

The mouse macrophage cell line RAW 267.4 (European Collection of Cell Cultures, Salisbury, UK) was routinely cultured in DMEM (GIBCO Invitrogen, Paisley, UK) supplemented with 10% heat-inactivated fetal calf serum (FCS: GIBCO Invitrogen), 100 U/mL penicillin, 100 mg/mL streptomycin, and 2 mM L-glutamine (DMEM/FCS). Histones and LDL were mixed and incubated for 1 hr at room temperature, before being added to sub-confluent RAW 267.4 cells in 24-well tissue culture plates. Cells were fixed and lipid content determined by Oil Red O staining[8]. Cytotoxicity was measured using a commercial kit based on lactate dehydrogenase (LDH) release, as directed by the manufacturer (Roche Diagnostics Ltd, Burgess Hill, UK). Optimal concentrations of histones and LDL, required for foam cell generation in the absence of cytotoxicity were determined empirically (data not shown). In subsequent experiments, RAW 267.4 cultures (n = 6) were incubated with: 50 µg/ml of LDL plus 50 µg/ml of histones; 50 µg/ml of LDL alone; 50 µg/ml of histones alone; vehicle. Following microscopic examination of Oil Red O staining, wells were washed with deionised water and air dried, then bound dye was solubilised by the addition of 250 µl of isopropanol. The concentration of solubilized Oil Red O determined by absorbance at 492 nm in a microplate reader, by reference to a standard curve.

## Results and Discussion

**Figure 1:**
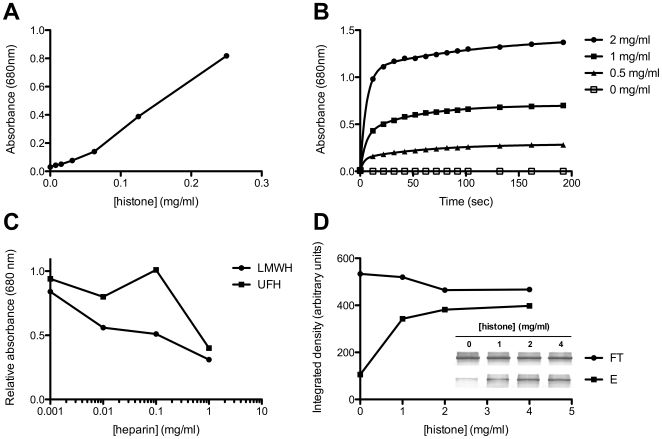
The influence of heparin on histone induced aggregation of low-density lipoprotein. **A:** Titration of histones into LDL (final concentration 1.25 mg/ml by protein) was carried out for 1 hr in PBS at 21°C, and protein aggregation measured by spectrophotometric absorbance at 680 nm. **B:** Rapid aggregation of LDL was observed over the first 180 seconds of incubation of LDL (75 µl of 1.33 mg/ml by protein in PBS) with histones (25 µl of 0, 0.5, 1 and 2 mg/ml in PBS). **C:** Inclusion of varying concentrations of low molecular weight heparin (LMWH) and unfractionated heparin (UFH) with LDL (1.25 mg/ml) and histone (0.25 mg/ml) caused a general decrease in protein aggregation, relative to uninhibited incubations. At 0.1 mg/ml, UFH was not effective. **D:** LDL (100 µl of 0.5 mg/ml in PBS) was incubated with 100 µl of heparin agarose slurry, to which 100 µl of 0, 1, 2 or 4 mg/ml calf thymus histones in PBS had previously been bound. The apolipoprotein B content in the unbound (FT) and SDS-eluted (E) fractions was determined by SDS-PAGE (inset) and quantified by densitometry. This indicated that LDL was selectively pulled down onto histone-charged heparin agarose. This experiment was performed three times with similar results.

To confirm the previously reported phenomenon of histone-induced aggregation of lipoproteins[6], human low-density lipoprotein (LDL) was titrated with calf thymus histones, an unfractionated mixture of histones H1, H2A, H2B, H3 and H4. This produced a concentration and time-dependent increase in turbidity (Figure 1A,B). Pre-treatment of LDL with LMWH gave a concentration-dependent inhibition of aggregation on addition of histones (Figure 1C). Unfractionated heparin gave a similar result at low concentrations of heparin, but at 0.1 mg/ml it was much less effective than LMWH (Figure 1C).

It is believed that proteoglycans such as perlecan, which are rich in the sulphated glycosaminoglycan heparan sulphate, play an important role in the retention of lipoproteins to endothelial extracellular matrix. Such proteoglycans are central to the “response to retention hypothesis” of atherosclerosis, proposed by Williams and Tabas[9]. Indeed, ApoE knockout mice which were also deficient in perlecan-associated heparan sulphate were found to be protected from development of atherosclerosis, compared to heparan sulphate sufficient controls[10]. In order to test whether histones were capable of performing a bridging role in the aggregation of LDL, we bound calf thymus histones to heparin agarose beads, where heparin was used as a structurally similar (but more highly sulphated) analogue of heparan sulphate[11]. Thus, if the bound histones were capable of pulling down LDL from solution, this would be consistent with a bridging (crosslinking) function. As shown in Figure 1D, a level of baseline adherence of LDL to heparin-agarose was observed, in accordance with the known affinity of apolipoprotein B for heparin[12]. With increasing levels of histones pre-bound to the heparin-agarose beads, more LDL was bound from solution, and recovered following elution of the beads with SDS-containing buffer. This indicates that histones non-covalently linked to a sulphated glycosaminoglycan-containing matrix under physiological conditions of pH and salt concentration are capable of binding LDL, suggesting a similar mechanism for lipoprotein retention to that proposed for lipoprotein lipase[13]. Although these model experiments conveniently used heparin, it has been shown that histones H1, H2A and H2B also have a high binding affinity for heparan sulphate[14], [15], [16]. Therefore, we suggest that histones may mediate bridging of LDL to heparan sulphate proteoglycans.

**Figure 2:**
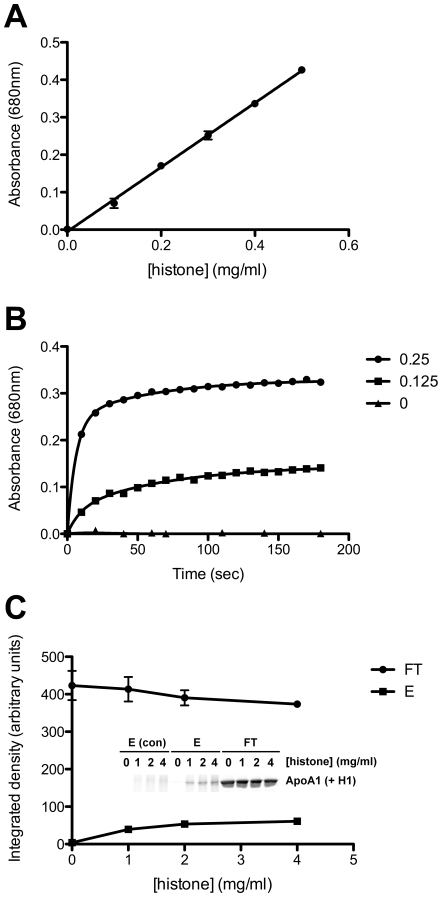
The influence of heparin on histone induced aggregation of high-density lipoprotein. A: Titration of histones into HDL (final concentration 1 mg/ml by protein) was carried out for 1 hr in PBS at 21°C, and protein aggregation measured by spectrophotometric absorbance at 680 nm. B: Aggregation of HDL was observed over the first 180 seconds of incubation of HDL (50 µl of 1 mg/ml by protein in PBS) with histones (50 µl of 0, 0.5 and 1 mg/ml in PBS). C: HDL (100 µl of 1 mg/ml in PBS) was incubated with 100 µl of heparin agarose slurry, to which 100 µl of 0, 1, 2 or 4 mg/ml calf thymus histones in PBS had previously been bound. The ApoA1 content in the unbound (FT) and SDS-eluted (E) fractions was determined by SDS-PAGE (inset) and quantified by densitometry (see Materials & Methods). Graph shows mean ± SEM for an experiment run in duplicate. This indicated that HDL was selectively pulled down onto histone-charged heparin agarose. This experiment was performed twice with similar results.

To determine whether the phenomenon of histone-induced lipoprotein aggregation was limited to LDL, additional experiments were carried out with HDL (Figure 2). This indicated a concentration and time-dependent aggregation of HDL in the presence of histones (Figure 2A and 2B, respectively). Furthermore, like LDL, HDL was selectively pulled down from solution by histone-charged heparin agarose (Figure 2C). Therefore LDL and HDL particles, which differ markedly in size, density and apolipoprotein content, nevertheless both interact with histones in an apparently similar manner.

**Figure 3:**
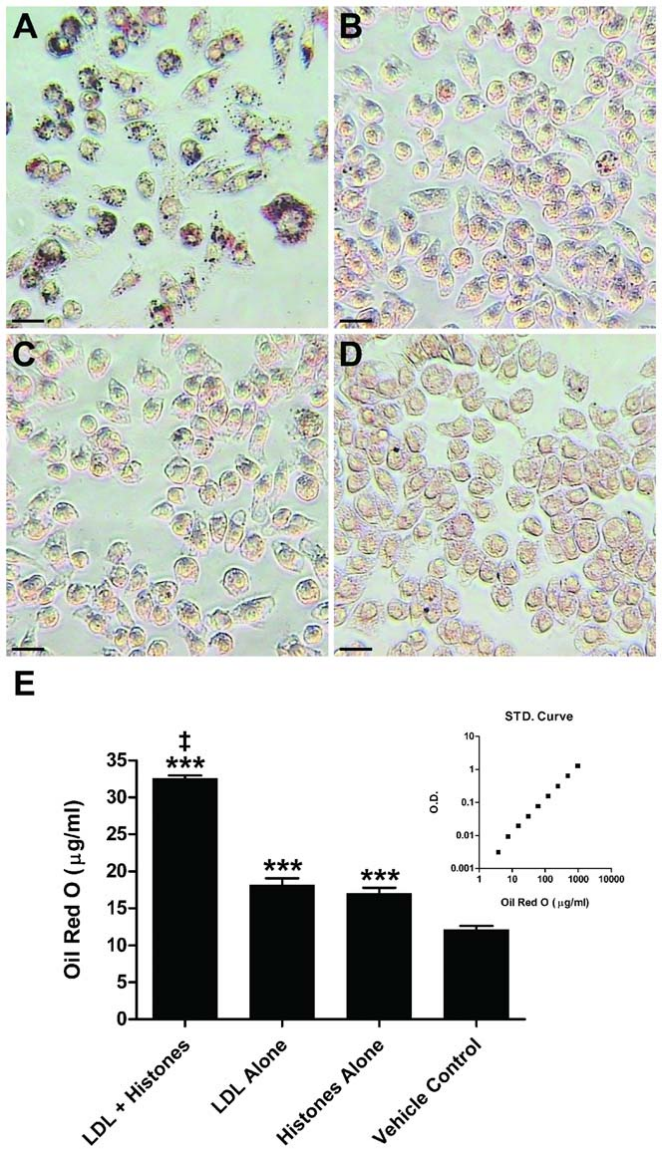
Histone-aggregated LDL is preferentially taken up by macrophages. Lipid uptake by RAW 264.7 macrophages was visualised by Oil Red O staining following incubation for 24 hr with **A:** LDL (50 µg/ml) plus histones (50 µg/ml), **B:** LDL alone, **C:** histones alone, or **D:** vehicle. **E:** The plate was then washed and dried, and Oil Red O solubilised from adherent cells by addition of isopropyl alcohol (250 µl per well). The concentration of cell-retained Oil Red O solubilised in this way was determined by absorbance at 492 nm in a microplate reader, by reference to a standard curve (inset). All treatments resulted in significantly greater lipid uptake by RAW cells compared to vehicle control (***, p<0.001). Incubations with LDL in the presence of histones induced a significantly greater lipid uptake than with LDL alone or histones alone (‡, p<0.001). This was repeated three times with similar results.

Since the formation of lipid-laden macrophages (foam cells) is a hallmark of the formation of the atherosclerotic plaque, we also investigated the influence of histones on the accumulation of LDL by a mouse macrophage line. RAW macrophage cells cultured for 24 hr with LDL (50 µg/ml by total apolipoprotein) contained more lipid, as visualised by Oil Red O histochemistry (Figure 3A-D), when histones (50 µg/ml) were present. The bound dye was solubilised using isopropanol and quantified by spectrometry, which indicated significantly enhanced lipid binding in the case of LDL plus histone treatment, compared to LDL or histone alone (Figure 3E). Histones also significantly increased lipid accumulation by RAW cells in the absence of exogenous LDL, presumably by enhancing uptake of endogenous lipoproteins from the FCS in the culture medium. There was no evidence for significant differences in cell viability between any of the different treatments.

Whilst the physicochemical properties of histones discussed above are consistent with a potential pro-atherosclerotic function, an important question remains as to how histones could possibly come into contact with the endothelial extracellular matrix. Possibilities may include lysis of cells within the endothelium, or transport of histones in complex with lipoproteins or other plasma proteins from remote areas.

Since histones have been found to be present at low levels in normal human plasma[17], it would be interesting to determine whether circulating histone levels are increased during chronic inflammation, as this may contribute to the known association between inflammation and atherosclerosis[18]. Chronic inflammatory conditions such as rheumatoid arthritis and systemic lupus erythematosus are associated with increased incidence of cardiovascular disease[19]. Furthermore, in models of sepsis, acute inflammation leads to the release of highly elevated levels of extracellular histones in the circulation, which mediate organ failure and death[20]. We therefore hypothesise that chronic inflammation may likewise result in elevated, although not acutely toxic, levels of histones entering the circulation. While this has not been addressed directly, a proteomic study of rheumatoid arthritis for example, reported that histone H2B was detected in arthritic but not control plasma[21].

The potential involvement of extracellular histones in atherogenesis may be envisaged through several routes. Firstly, lipoproteins such as LDL to which histones have become attached may have a higher affinity for binding to endothelial proteoglycans, and may thereby lead to enhanced lipoprotein retention. In addition, the secondary necrosis of foam cells, which is known to occur at the periphery of the necrotic or lipid core of the atherosclerotic lesion[22], would lead to local extracellular histone release, enhanced lipoprotein aggregation and in turn, enhanced uptake by further macrophages, in a positive-feedback mechanism leading to accelerated progression of the lesion.

Thus, we suggest that extracellular histones are worthy of further investigation in terms of their atherogenic potential. It will be important to discover how individual histone types contribute to this phenomenon, since this study used unfractionated histones as proof of concept. The affinity of histones for binding lipoprotein classes other than LDL and HDL should also be explored.

In conclusion, we have demonstrated *in vitro* properties of histones that are consistent with a potential pro-atherogenic role. If confirmed by *in vivo* studies, this could represent an important new target for the treatment of cardiovascular disease, particularly disease associated with chronic inflammatory conditions.
